# MIF promotes Th17 cell differentiation in rheumatoid arthritis through ATF6 signal pathway

**DOI:** 10.1186/s10020-024-01005-4

**Published:** 2024-11-29

**Authors:** Guozhi Yan, Rongrong Song, Jieyu Zhang, Zhihao Li, Zhantao Lu, Zijian Liu, Xiaokang Zeng, Jie Yao

**Affiliations:** 1 Department of Laboratory Medicine, The Sixth Affiliated Hospital, School of Medicine, South China University of Technology, Foshan, Guangdong 528200 China; 2 Central Laboratory of The Sixth Affiliated Hospital, School of Medicine, South China University of Technology, Foshan, Guangdong 528200 China; 3 The Department of Laboratory Medicine, Shunde Hospital, Southern Medical University, The First People’s Hospital of Shunde Foshan, Foshan, Guangdong China; 4 Department of Laboratory Medicine, Sun Yat-Sen Memorial Hospital, Sun Yat-Sen University, Guangzhou, Guangdong China

**Keywords:** Rheumatoid arthritis, MIF, ER stress, ATF6, Th17 cell

## Abstract

Rheumatoid arthritis (RA) is a common autoimmune disease that can lead to irreversible joint damage when it occurs, but its pathogenesis has not yet been elucidated. In this study, we explored the roles of macrophage migration inhibitory factor (MIF), endoplasmic reticulum stress (ER stress), and Th17 cells in the pathogenesis of RA. We have preliminarily confirmed that MIF expression in CD4^+^T cells and the proportion of Th17 cells are increased in active RA patients. We also found that ER stress is activated, initiating ATF6 pathway in the UPR. Additionally, using in vitro stimulation and co-immunoprecipitation experiments, we have confirmed the interaction between MIF and ATF6, which enhances protein expression in ATF6 pathway. Subsequently, in the chromatin immunoprecipitation assay, we observed the enrichment of ATF6 subunit on the promoter sequences of the Th17 cell differentiation genes STAT3 and RORC. Additionally, the differentiation of Th17 cells was disrupted by Ceapin-A7 (ATF6 inhibitor). In summary, our results indicate that MIF enhances ATF6 pathway signaling, which promotes the differentiation of Th17 cells. This could be a potential mechanism underlying the pathogenesis of RA, offering a new direction for the clinical treatment of RA.

**Supplementary Information:**

The online version contains supplementary material available at 10.1186/s10020-024-01005-4.

## Introduction

RA is one of the most common systemic autoimmune diseases, predominantly affecting women and capable of onset at any age, with a higher incidence between the ages of 40 and 70 (Smolen et al. 2016). Its clinical symptoms include joint pain, stiffness, swelling, and tenderness (Grassi et al. 1998). According to the Global Burden of Disease (GBD) report, from 1990 to 2017, the global prevalence of RA increased by 7.4%, and the incidence rate also increased by 8.2% (Finckh et al. 2022). In China, the prevalence of RA was 0.28% in 2012, which was higher than that in 2008 (Li et al. 2012). In the early stage of the disease, synovitis can cause joint damage. If not treated in time, it can lead to irreversible joint damage and in severe cases, it can affect other organs, causing diseases such as pericarditis, bronchiectasis, and pulmonary fibrosis (Figus et al. 2021). The pathogenesis of RA is very complex, related to genetics, immunity, and other factors, and has not been fully elucidated yet. Therefore, it is necessary to further explore the pathogenesis of RA and search for effective treatment strategies to alleviate the suffering of patients.

The imbalance of Th17/Treg caused by elevating proportion of Th17 cells is one of the factors contributing to the onset and progression of RA (Noack and Miossec 2014). Th17 cells dominate and secrete large amounts of IL-17 A, thereby enhancing the development of inflammation, which is observed in autoimmune diseases such as RA and HT (Hirota et al. 2007; Xue et al. 2015a, b). MIF is an upstream factor for many pro-inflammatory factors and promotes the occurrence of inflammation. Mikulowska A et al. found that MIF is a key mediator in RA (Mikulowska et al. 1997). Additionally, recent studies also indicate that the upregulation of MIF in autoimmune diseases is associated with the severity of the disease, and the use of MIF monoclonal antibodies can inhibit the inflammatory response (Leech et al. 1999). Xue et al. found that serum MIF was positively correlated with IL-17 A in patients with Hashimoto’s thyroiditis, and MIF was positively correlated with the proportion of Th17 cells (Xue et al. 2015a, b). However, the mechanism by which MIF affects Th17 cells and contributes to the pathogenesis of RA is not yet clear, and this is a subject worth exploring further.

ERS is a key factor in the pathogenesis of many autoimmune diseases (Feldmann et al. 1996; Bettigole and Glimcher 2015). A high concentration of inflammatory factors affects protein folding within cells. When unfolded proteins accumulate to a certain level, it triggers ERS and initiates the UPR (Sammels et al. 2010). The occurrence of UPR plays a significant role in the development and functional regulation of T cells (Kemp et al. 2019). Therefore, maintaining the stability of the endoplasmic reticulum is crucial for cell survival and normal function.

In this study, we confirmed that ATF6 pathway was activated and the expression of MIF was elevated in CD4^+^T cells of active RA patients. Additionally, MIF interacted with ATF6, enhancing the signaling of ATF6 pathway, which in turn promoted Th17 cell differentiation. Our research provides evidence for the potential pathogenesis of RA and offers new insights for the treatment of RA.

## Materials and methods

### Subject

Patients with RA treated at Shunde Hospital affiliated with Southern Medical University were randomly included in the study. Patients were included based on the 2010 American College of Rheumatology and European League Against Rheumatism (ACR/EULAR) criteria for diagnosis, confirming them as RA patients who were not within the clinical remission criteria (DAS28-ESR>2.6), and those with other autoimmune diseases were excluded (Felson et al. 2011). Additionally, patients with recent infections or other conditions such as tumors and infectious diseases were also excluded. Finally, 15 patients were selected for the study. At the same time, 15 healthy volunteers, matched for gender and age, with no diseases or infections, were recruited to serve as a healthy control group (HC). Ethical approval for this study was granted by the Ethics Committee of Shunde Hospital of Southern Medical University. The clinical conditions of the patients are presented in Table 1.

**Table 1:** Clinical characteristics of the patients and normal control subjects

	HC	RA	p value	Normal range
Case(n)	15	15	-	-
Gender(F/M)	14/1	14/1	-	-
Age(years)^a^	52.8 ± 5.23	53.27 ± 5.46	0.8127^c^	-
RF(IU/ml)^b^	4.3 (0.8–7.9)	92.2 (36.8–225.0)	< 0.0001^c^	0–20
Serum anti-CCP(U/ml)^b^	1.5 (0.4–2.8)	157.9 (55.3–253.0)	< 0.0001^c^	0–5
CRP(MG/L)^b^	1.2 (0.1–3.4)	19.3 (9.1–36.9)	< 0.0001^c^	0–6
ESR(mm)^b^	6.0 (2.1–10.8)	34.7 (19.2–55.8)	< 0.0001^c^	0–15
DAS28-ESR^b^	-	5.9 (4.0-6.9)	-	-

HC, Healthy control; RA, Rheumatoid arthritis; F, female; M, male; RF, Rheumatoid factor; Serum anti-CCP, Serum Anti-cyclic citrullinated peptide; CRP, C-reactive protein; ESR, erythrocyte sedimentation rate; DAS28-ESR, Disease Activity Score 28-ESR

^a^ Data provided in mean ± SD

^b^ Data provided in median (p5th-p95th)

^c^ Representative p value in HC vs. RA

### PBMC isolation and CD4+T cell sorting

Peripheral blood mononuclear cells (PBMCs) were isolated from heparinized blood using the Lymphoprep™ density gradient centrifugation method for cell sorting. The separation medium was purchased from STEMCELL (Canada).

The collected PBMCs were subjected to negative selection using the Human CD4^+^T Cell Isolation Kit (STEMCELL, Canada). The purity of the isolated cell population was assessed by flow cytometry, and the cells were used for subsequent experiments only if the purity exceeded 90%. The viability of the cells was also determined using Trypan Blue staining.

### Cell culture

PBMCs were cultured in RPMI1640 medium supplemented with 1% antibiotics (Gibco, UK) and 10% fetal bovine serum (Gibco, UK), and incubated in a 37 °C, 5% CO2 incubator.

#### ELISA

According to the manufacturer’s instructions (Neobioscience, China), an ELISA kit for human IL-17 A was used to measure the concentration of IL-17 A in the serum samples of volunteers by enzyme-linked immunosorbent assay. The detection range of the kit is 6.25–400 pg/ml.

### Western blot analysis

To the purified CD4^+^T cells, a cell lysis buffer containing PMSF (Beyotime, China) was added, and the cells were lysed on ice. The lysate was then centrifuged at 4 °C, and the supernatant was collected. The proteins were mixed with a loading buffer (5x, EpiZyme, China) and heated at 37 °C for 30 min, then subjected to SDS-PAGE followed by transferring onto PVDF membrane for immunoblotting analysis. The antibodies used are as follows: ATF6, BIP, XBP1s, ATF4 and GAPDH purchased from Proteintech (China), RORC and STAT3 purchased from HUABIO (China).

### Real-time PCR

According to the manufacturer’s protocol, total RNA was extracted from CD4^+^T cells using the Total RNA Kit (Mabio, China). The mRNA was reverse transcribed into cDNA using the HiScript II Q RT SuperMix for qPCR kit, and the expression of target genes was measured using the ChamQ SYBR qPCR Master Mix kit. The data were normalized to β-actin and transformed using relative quantification (2-ΔΔCt). All primer sequences are shown in Table 2.

**Table 2:** List of primer sequences

Gene (Human)	Forward primer (5′–3′)	Reverse primer (5′–3′)
**MIF**	CCCGGACAGGGTCTACATCA	GGAGTTGTTCCAGCCCACAT
**IL-17A**	TGATTGGAAGAAACAACGATGACT	ATTGTGATTCCTGCCTTCACTATG
**BIP**	CATCACGCCGTCCTATGTCG	CGTCAAAGACCGTGTTCTCG
**ATF6**	GACAGTACCAACGCTTATGCC	CTGGCCTTTAGTGGGTGCAG
**XBP1**	CCCTCCAGAACATCTCCCCAT	ACATGACTGGGTCCAAGTTGT
**ATF4**	CTCCGGGACAGATTGGATGTT	GGCTGCTTATTAGTCTCCTGGAC
**RORC**	AGAACACCATCTCCAGCCTCA	GAAGTCCTTAAATCCCAGCCAC
**STAT3**	GCTCTCAACCTCGCCACC	CGGAATGTCCTGCTGAAAAC
**β-Actin**	TGATGGTGGGAATGGGTCAGAA	CCAAGAAGGAGGGCTGGAAAG

### Co-immunoprecipitation assay

For the co-immunoprecipitation experiment, we used a co-immunoprecipitation kit (Thermo Fisher Scientific, USA) to co-precipitate proteins. We collected 1 × 107 CD4^+^T cells, lysed them with IP lysis buffer to extract the protein lysate. The resin coupled with specific antibodies ATF6 (CST, USA), MIF (Santa Cruz Biotechnology, USA), or IgG negative control (Beyotime Biotechnology, China) was mixed with the protein lysate in a shaker at 4 °C overnight. Then, The samples were washed multiple times with buffer, followed by elution with an elution buffer to elute the immunoprecipitated complexes for further Western blot analysis.

### Chromatin immunoprecipitation (ChIP) assay

The sorted CD4^+^T cells were collected into a centrifuge tube. Next, the cell fixed with formaldehyde at ambient temperature for 10 min for generating DNA–protein crosslink. The reaction was terminated by 10× Glycine Solution (final proportion of 1×). The samples were washed twice with PBS containing PMSF. The samples were lysed using SDS lysis buffer, sub-packed (200 µL per tube), and fragmented by ultrasonic wave, 4 s each at an interval of 3 s, 5 min in total. The supernatant was harvested through centrifugation at 14,000 rpm and 4ºC for 5 min. The supernatant were diluted using CHIP dilution buffer, mixed well, and sub-packed into 2 tubes. The tubes were respectively added with human antibody to IgG (Beyotime Biotechnology, China) as NC and human antibody to ATF6 (Proteintech, China) for incubation at 4ºC overnight. The endogenous DNA–protein complex was precipitated by Protein Agarose/Sepharose. The non-specific complex was washed and the DNA–protein complex was incubated at 65ºC for 6 h to reverse the crosslinks. DNA fragment was purified and retrieved by PCR/DNA purification kit (Beyotime Biotechnology, China). The primer was designed by selecting 2000 bp from the transcription start site to the upstream as the promoter sequence. The enrichment of ATF6α in the STAT3 and RORC promoter region was tested by RT-qPCR. All primer sequences are shown in Table 2.

### Flow cytometry

The proportion of Th17 cells was evaluated using anti-CD3-PC-cy5.5, anti-CD8-FITC, and anti-IL-17A-BV421(BD Biosciences, USA). 1× Cell Stimulation Mix (00-4975-93, Thermo Fisher Scientific, USA) was added to the cells and cultured at 37 °C for 6 h before collecting the cells. The cells were resuspended in buffer containing anti-CD3 and CD8, and incubated at 4 °C for 45 min. After washing, a fixation and permeabilization reagent was added and incubated at 4 °C for 20 min. Following another wash, the cells were resuspended in buffer containing anti-IL-17A and incubated at 4 °C overnight. The next day, the cells were washed and resuspended. Data were acquired on the BD AccuriTM C6 plus instrument (BD Biosciences, USA) and analyzed using FlowJo software.

### Cell stimulation experiment

Regarding the Tunicamycin™ stimulation experiment, TM (5 µg/ml, ACMEC, China) was added to PBMCs, and after 1 h of culture, the cells were sorted and protein lysates were extracted for subsequent experiments. For the recombinant human MIF (rhMIF) stimulation experiment, rhMIF (200ng/ml, MCE, USA) was added to PBMCs, and after 6 h of treatment, the cells were sorted and protein lysates were extracted for experimental use. rhMIF (200ng/ml, MCE, USA) was added to PBMCs for 3 days of treatment before flow cytometry analysis. For ATF6 inhibition assay, cells were treated with Ceapin-A7 (6µM, MCE, USA) for 1 h, then rhMIF (200ng/ml) was added to both the control and experimental groups to stimulate the cells for 6 h or 3 days, respectively, after which protein lysates were extracted and flow cytometry was performed.

### Statistical analysis

Differences between groups were analyzed by Mann-Whitney U test for non-paired comparisons or by Student’s t-test for paired comparisons. Data are presented as mean ± SEM. The target protein bands were quantified by ImageJ software after grayscale conversion for statistical analysis. All statistical procedures and figures were performed using GraphPad Prism 8 or SPSS version 25 statistical software package. P value less than 0.05 was considered statistically significant.

## Results

### Expression of MIF and IL-17A in CD4^+T^ cells of active RA patients

**Fig. 1:**
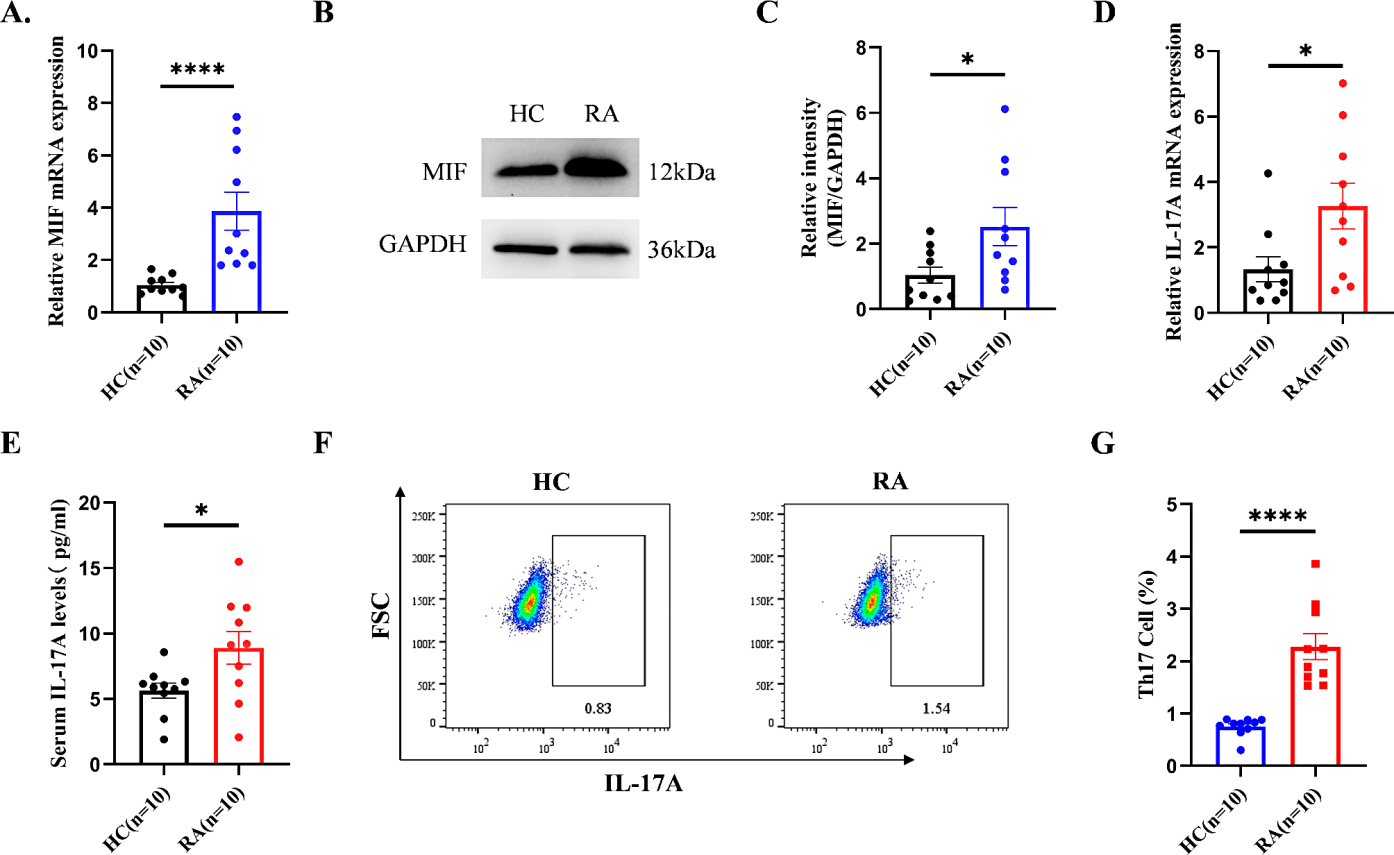
Expression of MIF and IL-17A in CD4^+^T cells of active RA patients. (**A** to **C**) Compared to healthy controls, MIF expression is increased at both mRNA and protein levels in RA. (**D** to **G**) Compared to healthy controls, IL-17A expression is increased at both mRNA and serum levels in RA. (**F**) Th17 cells were labeled with anti-CD3-PC-cy5.5, CD8-FITC, and IL-17 A-BV421 antibodies for flow cytometric analysis. HC, healthy controls; RA, rheumatoid arthritis. *p < 0.05, **p < 0.01, ***p < 0.001, ****p < 0.0001

After sorting CD4^+^T cells from RA and HC, we detected the expression of MIF and IL-17A using RT-qPCR, Western Blot, and ELISA. Compared to the healthy control group, MIF expression was significantly increased at the mRNA level in RA patients (Fig. 1A). Similarly, compared to the healthy control group, MIF expression was elevated at the protein level in RA patients (Fig. 1B, C). The trend of MIF expression was consistent at both the mRNA and protein levels. At the same time, compared to the healthy control group, IL-17A levels were significantly increased at both the mRNA and serum levels in RA patients (Fig. 1D, E). We then used flow cytometry to assess the proportion of Th17 cells in the peripheral blood of RA patients and healthy controls. The results showed that, compared to the healthy control group, the proportion of Th17 cells in RA patients was significantly increased (Fig. 1F, G), indicating that the appearance of Th17 cells upregulated the expression of IL-17A. These findings suggest that the upregulation of MIF is associated with the increased proportion of Th17 cells and the progression of RA.

### The ER stress in CD4^+^T cells of active RA patients is activated

**Fig. 2:**
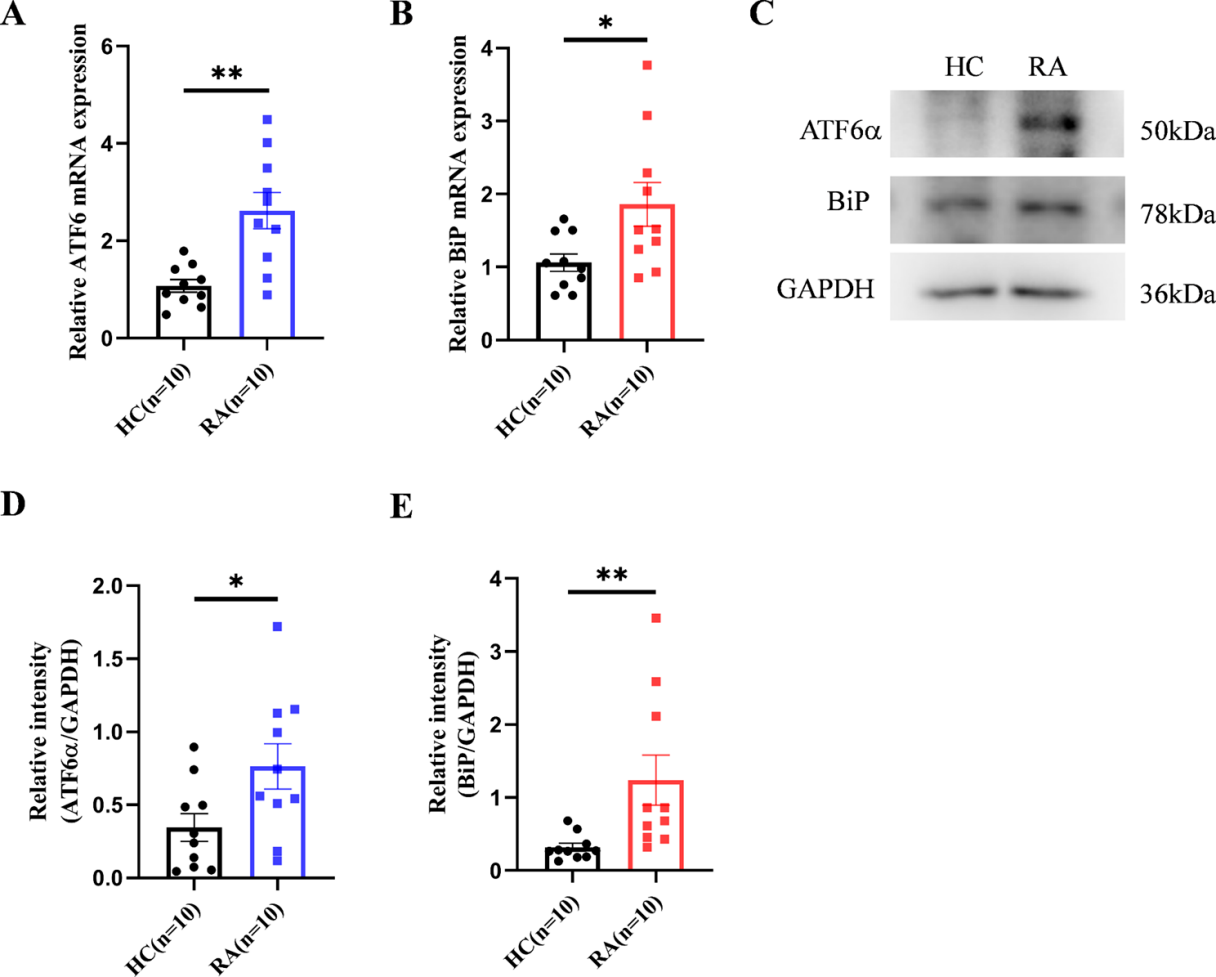
ER stress is activated in CD4^+^T cells of active RA patients. (**A**, **B**) RT-PCR detection of ATF6 and BiP mRNA expression. (**C**) Immunoblotting results show increased expression of ATF6α and BiP in RA compared to HC. (**D**, **E**) Quantitative analysis of the protein bands of ATF6α and BiP based on grayscale values, with GAPDH as the reference for statistical graphs. *p < 0.05, **p < 0.01

Endoplasmic reticulum homeostasis plays a crucial role in autoimmune diseases, affecting T cell function and differentiation (Kemp et al. 2019). Therefore, we detected the expression of multiple ER signaling pathway-related genes. The results showed that the expression of ATF6, XBP1and BiP increased at the mRNA level compared to healthy controls (Fig. 2A, B, supplementary Fig. 1B), while ATF4 did not show any changes (supplementary Fig. 1A). Correspondingly, we detected the protein levels. Compared to healthy controls, the expression of ATF6α and BiP was upregulated (Fig. 2C, D, E), while there were no changes in the expression of XBP1s and ATF4 (supplementary Fig. 1C, D). However, there were no significant differences in MIF levels and ER stress-related markers in CD4^+^T cells between RA patients in remission and healthy controls (supplementary Fig. 2). Our results confirmed that the ATF6 pathway of the UPR is activated in active RA patients. However, this phenotype was not observed in RA patients in remission.

### MIF interacts with ATF6 under stress conditions

**Fig. 3:**
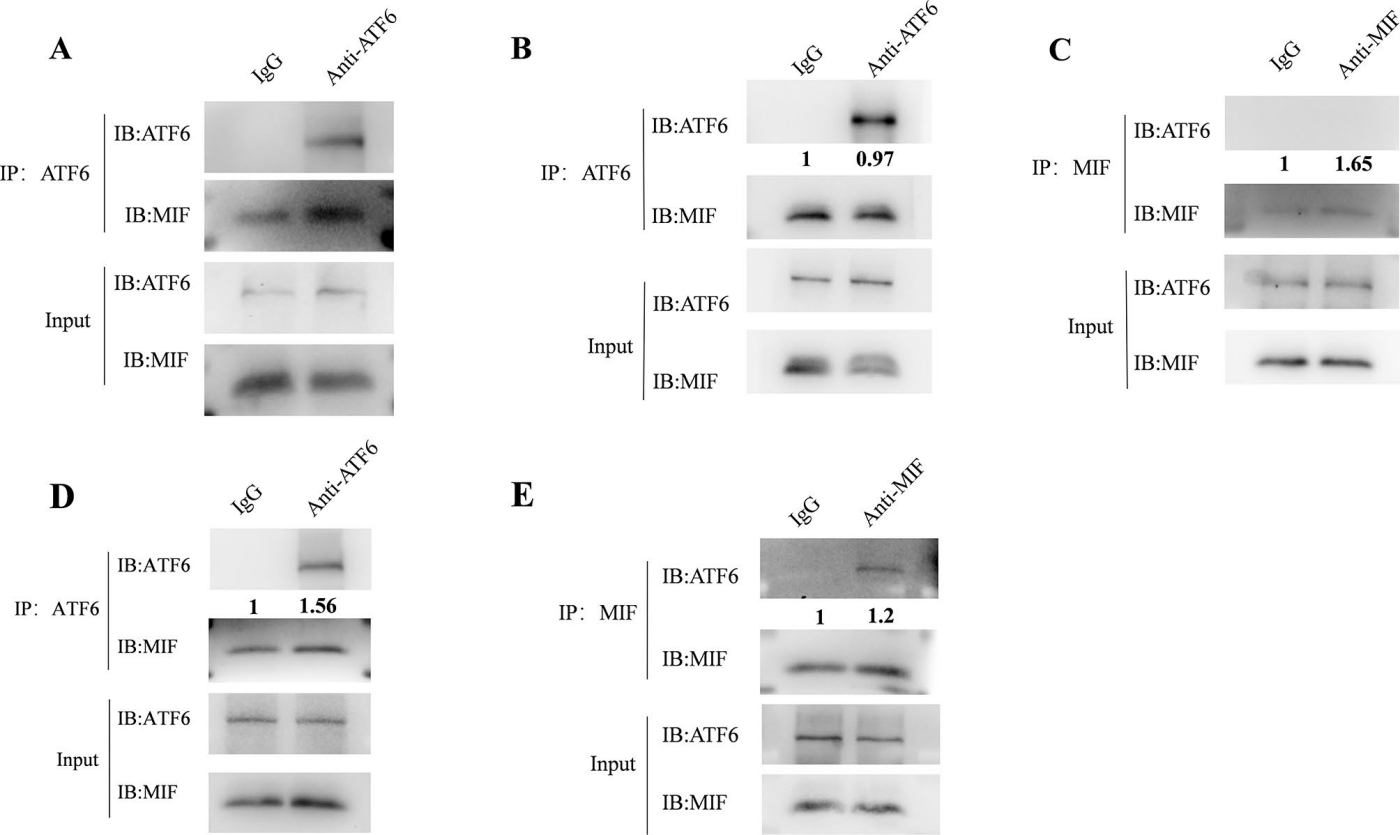
Interaction between MIF and ATF6. (**A**) Protein lysates were extracted from CD4^+^T cells of RA patients and used to isolate bait-prey protein complexes using ATF6 antibodies. (**B**,**C**,**D**,**E**) PBMCs from healthy controls were treated with TM for 1 h, sorted CD4^+^T cells, and extracted protein lysates for immunoprecipitation experiments. The results suggest that MIF interacts with ATF6 under ER stress conditions

Numerous studies have shown that ER stress is associated with the pathogenesis of autoimmune diseases, but the upstream signals leading to the occurrence of UPR have not yet been clearly studied (Barrera et al. 2018; Conza et al. 2023). MIF, on the other hand, is known to interact with multiple proteins to regulate cellular functions (Gore et al. 2008; Fex et al. 2017; Kim et al. 2017). Therefore, we speculate that MIF may bind to ATF6 to activate downstream signals. To confirm this speculation, we performed immunoprecipitation experiments on CD4^+^T cells from active RA patients, and the results showed that MIF and ATF6 interacted (Fig. 3A).

Based on the above results, we further speculate whether MIF is an upstream factor directly activating ATF6 pathway. To verify this, we conducted experiments on healthy human cells using tunicamycin (TM) to validate the occurrence of this process. Our results revealed that MIF was able to interact with ATF6 in the TM-treated group, whereas no interaction was observed in the untreated group (Fig. 3B, C, D, E). This suggests that the interaction between MIF and ATF6 occurs specifically under conditions of ERS.

**Fig. 4:**
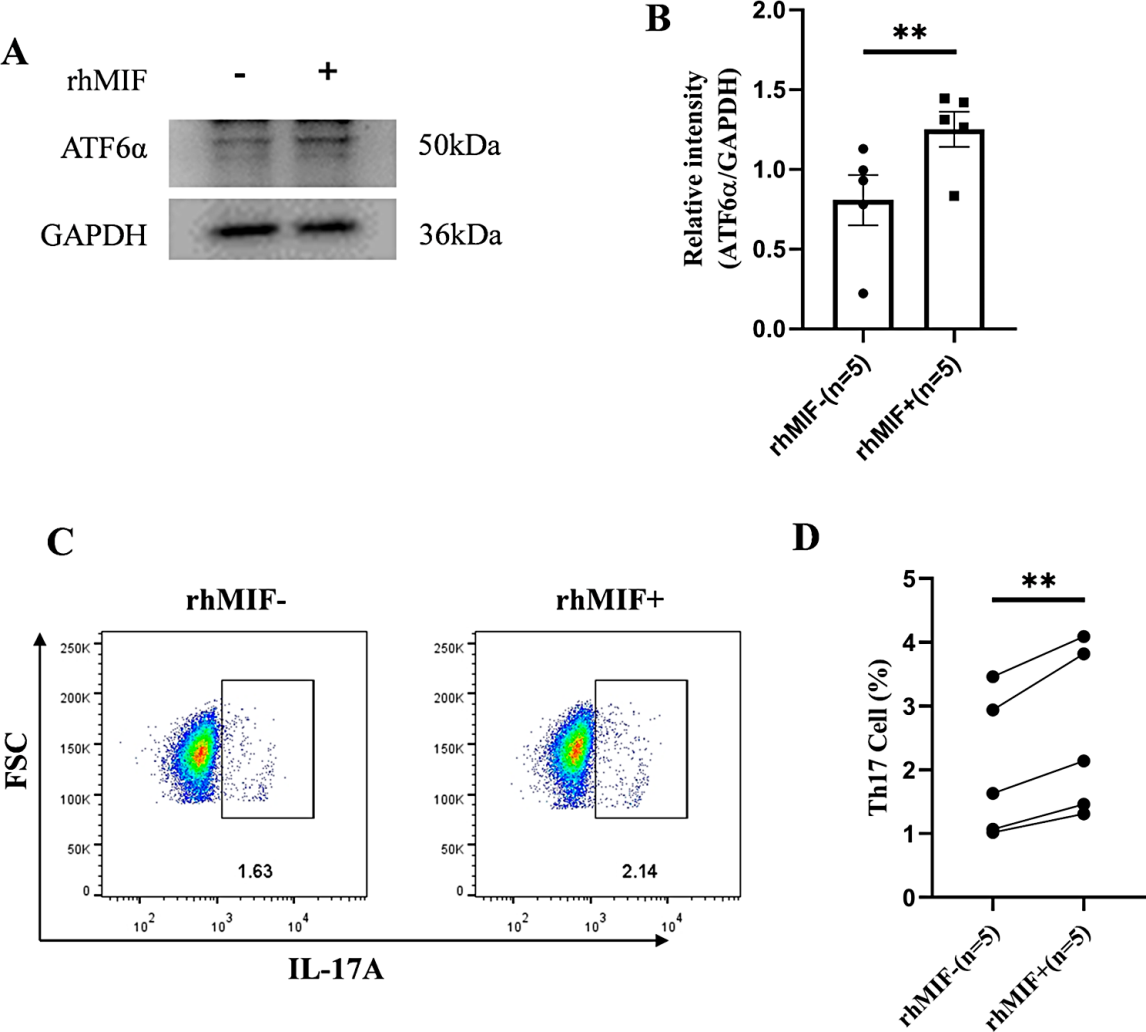
Elevated MIF enhances ATF6 pathway signaling and promotes Th17 cell differentiation. (**A**, **B**) rhMIF was treated with RA patient PBMCs for 6 h, sorted CD4^+^T cells, and extracted protein lysates for immunoblotting experiments. The protein band gray value was quantitatively analyzed and statistically. The protein level of ATF6α increased under rhMIF stimulation. (**C**, **D**) rhMIF was treated with RA patient PBMCs for 3 days, and flow cytometry was performed to analyze the proportion of Th17 cells in the control group and the treated group. The results showed that the proportion of Th17 cells increased in the treated group. *p < 0.05, **p < 0.01

Subsequently, we conducted an in vitro stimulation experiment using rhMIF on cells from RA patients. The results showed that rhMIF was able to upregulate the expression of ATF6α (Fig. 4A, B). At the same time, we performed flow cytometry on RA patient peripheral blood cells treated with rhMIF, and the results showed that the proportion of Th17 cells increased (Fig. 4C, D). Based on the above results, we propose that MIF acts as a regulatory factor of ATF6 pathway, enhancing its signaling response and promoting Th17 cell differentiation.

### Through ATF6 pathway, the differentiation genes of Th17 cells are induced to express

**Fig. 5:**
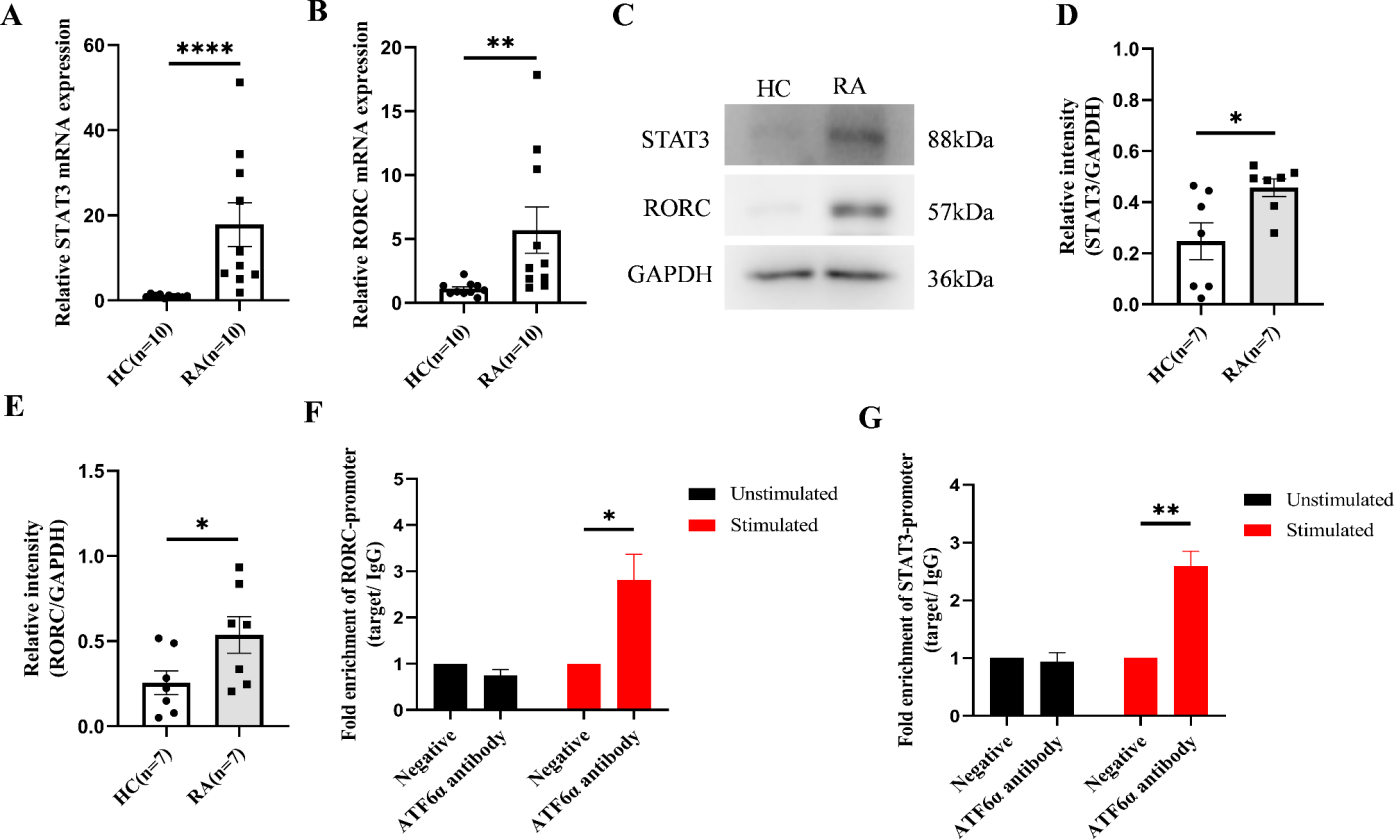
ATF6α regulates the transcription of Th17 cell differentiation genes STAT3 and RORC. (**A**, **B**). Compared to the healthy control group, the mRNA expression of STAT3 and RORC in CD4 + T cells of RA patients is upregulated. (**C**, **D**, **E**). At the protein level, the expression of STAT3 and RORC in RA patients is higher than that in the healthy control group. (**F**, **G**). Stimulated group, after treating PBMC1h with TM, CD4 + T cells were sorted and lysates were extracted for CHIP. Unstimulated group, no treatment was performed. Compared to the untreated group, the activated ATF6 pathway in the treated group resulted in ATF6α recovering more promoter sequences of STAT3 and RORC. TM, tunicamycin. *ρ < 0.05, **ρ < 0.01, ***p < 0.001, ****p < 0.0001

Our previous results showed an increased proportion of Th17 cells in RA patients. To further understand the role of enhanced ATF6 pathway signaling on Th17 cells, we examined the expression of Th17 cell differentiation-related genes STAT3 and RORC. These two genes play a crucial role in the differentiation process of Th17 cells, and the activation of ER stress is important in the differentiation of T cells (Kemp et al. 2019). Therefore, we first detected the expression of STAT3 and RORC in CD4^+^T cells from RA patients. The results showed that compared to the healthy control group, the expression of STAT3 and RORC in RA patients was upregulated at both the mRNA level and the protein level (Fig. 5A, B, C, D, E). Subsequently, we used CHIP assay to verify whether ATF6α was enriched on the promoters of STAT3 and RORC. The results showed that compared to the TM untreated group, ATF6α in the TM-treated group was able to recover more promoter sequences of STAT3 and RORC (Fig. 5F, G). This indicates that under stress conditions, ATF6α becomes enriched on the promoter sequences of STAT3 and RORC, initiating gene transcription and promoting the differentiation of CD4^+^T cells into Th17 cells.

### ATF6 inhibitor ceapin-A7 inhibits the differentiation of Th17 cells

**Fig. 6:**
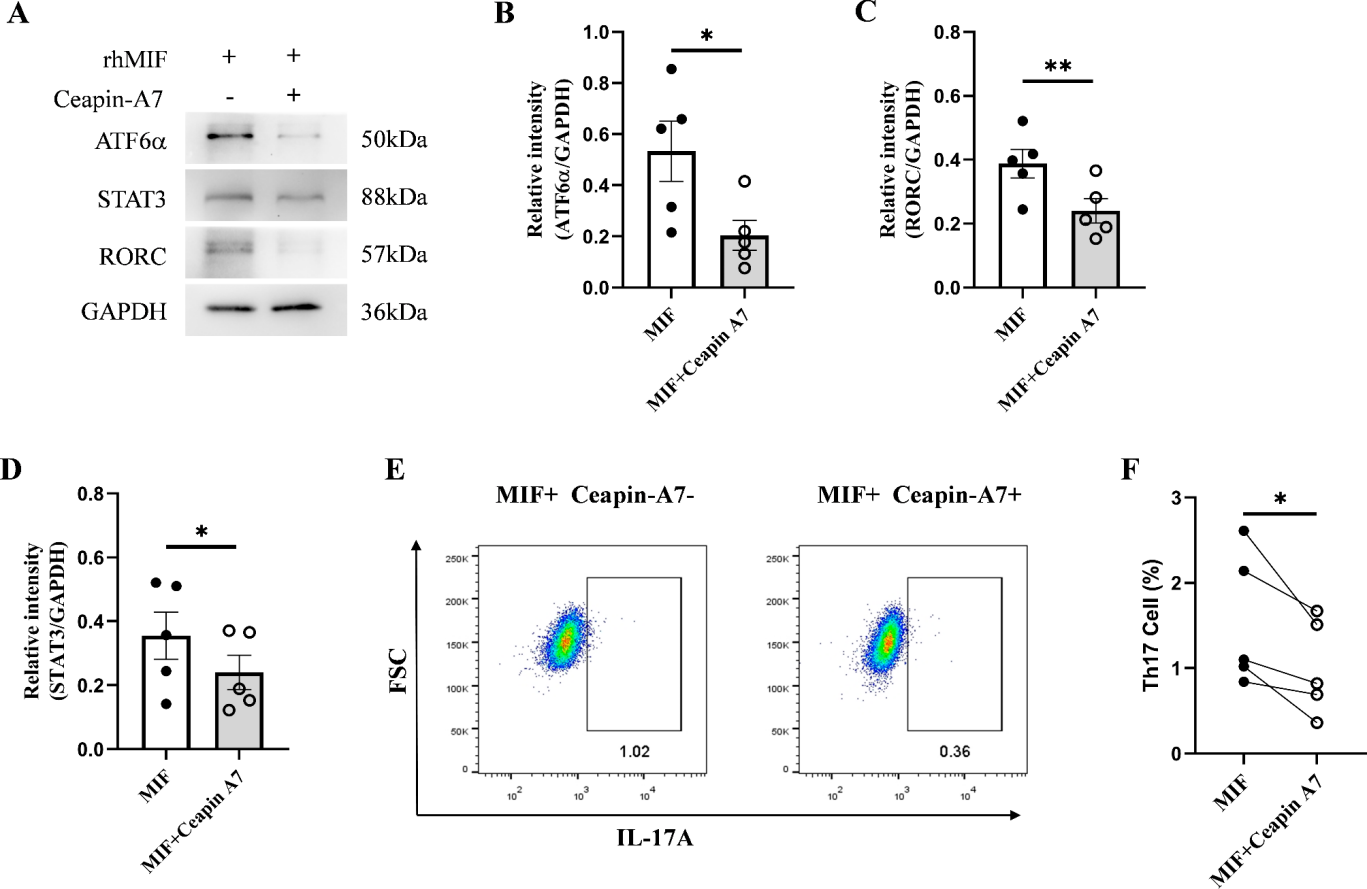
Ceapin-A7 inhibits the activation of ATF6 pathway and affects the differentiation of Th17 cells. (**A**). The expression of ATF6α, STAT3, and RORC in RA patient cells stimulated with rhMIF in the presence of Ceapin-A7. (**B**, **C**, **D**). The expression of ATF6α, STAT3, and RORC is reduced under the influence of Ceapin-A7. (**E**, **F**) After treating with Ceapin-A7 for 1 h and stimulating with rhMIF for 3 days, flow cytometry was used to analyze the effect of Ceapin-A7 on Th17 cells. *ρ < 0.05

To further verify that MIF induces the differentiation of Th17 cells through ATF6 signaling pathway, we used ATF6 inhibitor Ceapin-A7 to inhibit ATF6 signaling pathway. Our results showed that compared to rhMIF control group, the expression of ATF6α was inhibited in the presence of Ceapin-A7 (Fig. 6A, B), indicating that Ceapin-A7 can interfere with the cleavage of ATF6. Subsequently, we measured STAT3 and RORC and found that their expression was also inhibited after the suppression of ATF6 pathway (Fig. 6A, C, D), further indicating that STAT3 and RORC are targets of ATF6α. In addition, we found that the proportion of Th17 cells decreased in the presence of Ceapin-A7 (Fig. 6E, F).

## Discussion

Currently, patients with RA still require lifelong treatment, and the disease can reduce labor capacity. This not only brings physical pain to patients but also increases their economic burden. Therefore, understanding the underlying mechanisms of the disease provides a direction for the development of new treatment strategies in the future. This study attempts to reveal the role of MIF in RA. In our study, it was demonstrated that MIF can promote the differentiation of Th17 cells through ATF6 signaling pathway in active RA patients, accelerating the progression of RA.

MIF is a key mediator in various immune and inflammatory responses, including diseases such as RA and cancer (Mikulowska et al. 1997). Scholars both domestically and internationally have confirmed that the high expression of MIF and IL-17 A in the peripheral blood is related to the pathogenesis of diseases such as HT and SLE (Xue et al. 2015a, b; Ayaz et al. 2014). Our study shows that the mRNA and protein levels of MIF in CD4^+^T cells of active RA patients are upregulated, may indicating that MIF plays a role in the progression of RA. Studies have reported that the imbalance of Th17 cells is a key factor in the occurrence and development of RA (Hernandez-Palma et al. 2019), and the proportion of Th17 cells increases in autoimmune diseases (Liu et al. 2023), which is consistent with our results. The expression of IL-17A in the serum was also found to be increased. IL-17A is an important factor in the pathogenesis of various chronic inflammatory diseases, including RA and SLE. Studies have reported a positive correlation between MIF and Th17 cells in autoimmune diseases (Liu et al. 2023), and IL-17A expression is impaired in MIF knockout mice (Luo et al. 2021). These studies suggest that MIF is closely related to Th17 cells, but the molecular mechanisms by which MIF affects the imbalance of Th17 cells are not yet clear.

The endoplasmic reticulum has multiple biological functions, and maintaining ER stability is key to the survival and normal function of cells. In autoimmune diseases, a large number of inflammatory factors can affect protein folding within cells. When unfolded proteins accumulate to a certain level, it triggers ERS and initiates the UPR (Sammels et al. 2010). After the activation of ERS, it can further lead to the development of the disease, such as IRE1α activating the NF-κB pathway, resulting in the release of TNF-α and interleukins (Kaneko et al. 2003). Numerous studies have confirmed that ER stress is a key factor in the occurrence of autoimmune and inflammatory diseases. Therefore, we examined the expression of UPR-related genes and proteins and found increased expression of ATF6α and BiP in RA patient cells, suggesting UPR activation. Since inflammation is one of the factors activating UPR, can the immune factor MIF participate in UPR activation? Currently, the research on this aspect is not clear.

MIF is a multifunctional cytokine that can bind to CD74/CD44 outside the cell to regulate the immune response and has many functions inside the cell. It can interact with various proteins to regulate cell function (Gore et al. 2008; Fex et al. 2017; Kim et al. 2017). In the above results, it was found that the expression of MIF and ATF6 in the cells of RA patients increased, suggesting that MIF and ATF6 may interact and regulate cell function. To verify the relationship between MIF and ATF6, immunoprecipitation technology was used, confirming the interaction between MIF and ATF6 in RA patients. To further determine the role of MIF in ER stress, we used tunicamycin to treat normal human cells, and the results confirmed that the interaction between MIF and ATF6 occurs after ER stress. Additionally, we stimulated RA patient cells with rhMIF, and the results showed an increase in ATF6 protein expression. These results suggest that MIF acts as a promoter of the ATF6 pathway, enhancing ATF6 pathway signaling.

Previous studies and numerous literatures have indicated the close relationship between the increase in MIF and the imbalance of Th17 cell proportions. Therefore, we first verified that the expression of genes related to Th17 cell differentiation, STAT3 and RORC, was increased in CD4^+^T cells of RA patients. Then, we treated RA patient cells with rhMIF, and the flow cytometry results showed an increase in the proportion of Th17 cells. This suggests that MIF can induce the differentiation of Th17 cells. Based on these results, we speculated that MIF might promote the differentiation of Th17 cells through ATF6 pathway. ATF6 is a transcription factor that normally resides in an inactive state on the endoplasmic reticulum membrane. Upon activation, it dissociates from BiP and translocates to the Golgi apparatus, where it is hydrolyzed by the proteases SIP and S2P, forming the active ATF6α. This active form then enters the nucleus to exert its function as a transcription factor, and also upregulates the expression of proteins such as BiP, thereby restoring intracellular homeostasis (Shen et al. 2002; Haze et al. 1999). To verify whether ATF6α enriches at the promoters of STAT3 and RORC, we conducted a CHIP experiment which confirmed that ATF6α can bind to the promoter sequences of STAT3 and RORC, initiating transcription.

To further elucidate the regulation of MIF on Th17 cells, we used ATF6 inhibitor Ceapin-A7 to inhibit the production of ATF6α. The results showed that in the presence of Ceapin-A7 and rhMIF treatment, the expression of ATF6α in CD4^+^T cells was reduced in the Ceapin-A7 group, and the expression of RORC and STAT3 was also reduced. Flow cytometry results showed that compared to the control group, the proportion of Th17 cells in the Ceapin-A7 group was reduced. This further indicates that MIF upregulates the expression of STAT3 and RORC through the ATF6 signaling pathway, promoting the differentiation of Th17 cells.

In summary, we have confirmed that MIF plays an important role in the development of RA. High expression of MIF binds to ATF6, enhancing ATF6 signal, forming active ATF6α, which enters the nucleus to regulate the transcription of Th17 cell differentiation genes STAT3 and RORC, promoting the differentiation of Th17 cells and ultimately accelerating the progression of RA. This could be a potential mechanism underlying the pathogenesis of RA, offering a new direction for the clinical treatment of RA.

## Electronic supplementary material

Below is the link to the electronic supplementary material.

Supplementary Material 1

Supplementary Material 2

## Data Availability

The data used to support the findings of this study are available from the corresponding author upon request.
