# Supplementary figures and images for: MIF promotes Th17 cell differentiation in rheumatoid arthritis through ATF6 signal pathway
Source: Mol Med. 2024 Nov 29;30:237. doi: 10.1186/s10020-024-01005-4 (PMC11605992; doi:10.1186/s10020-024-01005-4)

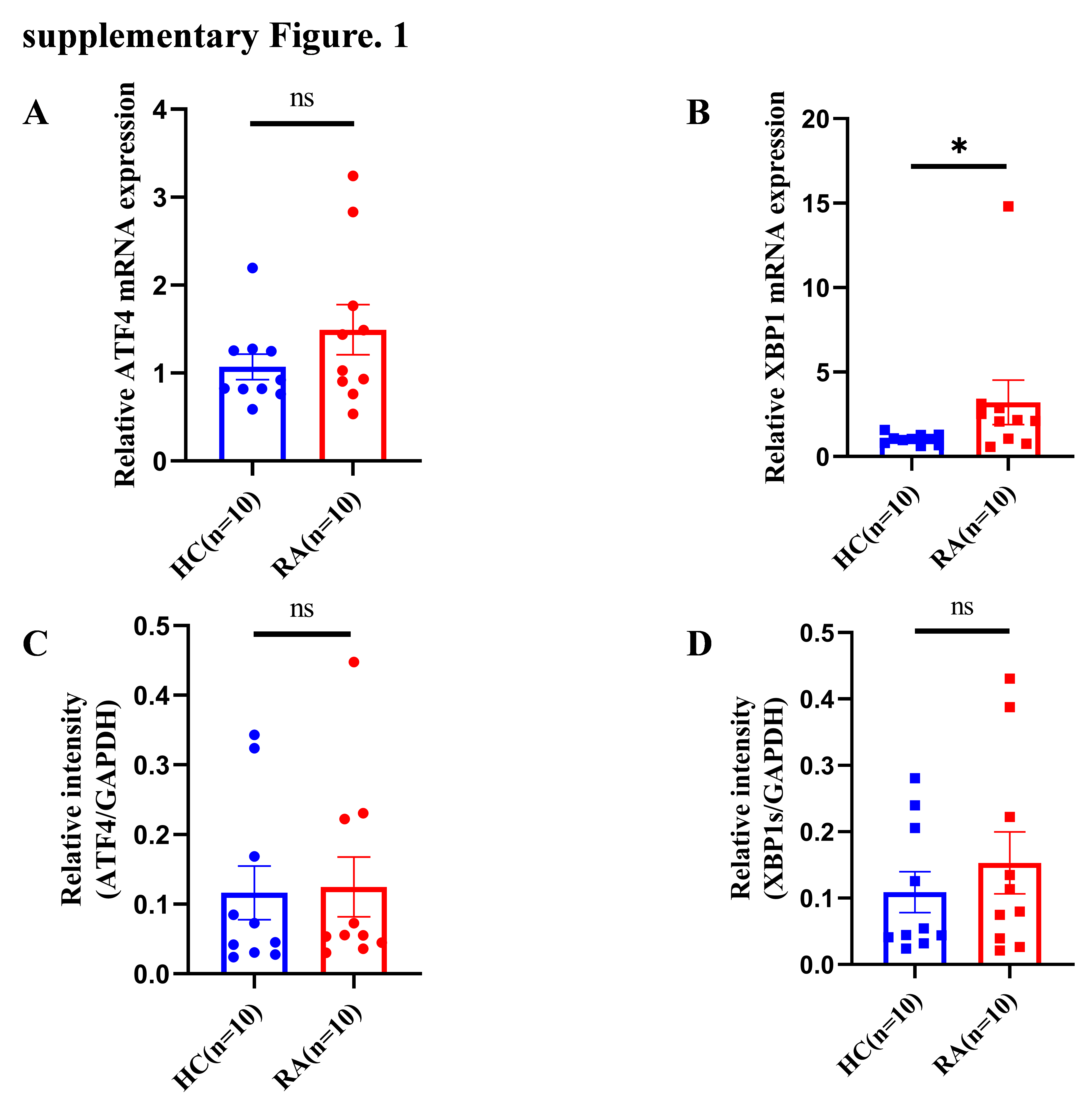

Supplement: Supplementary file 1 — Supplementary Material 1 [file 10020_2024_1005_MOESM1_ESM.tif]

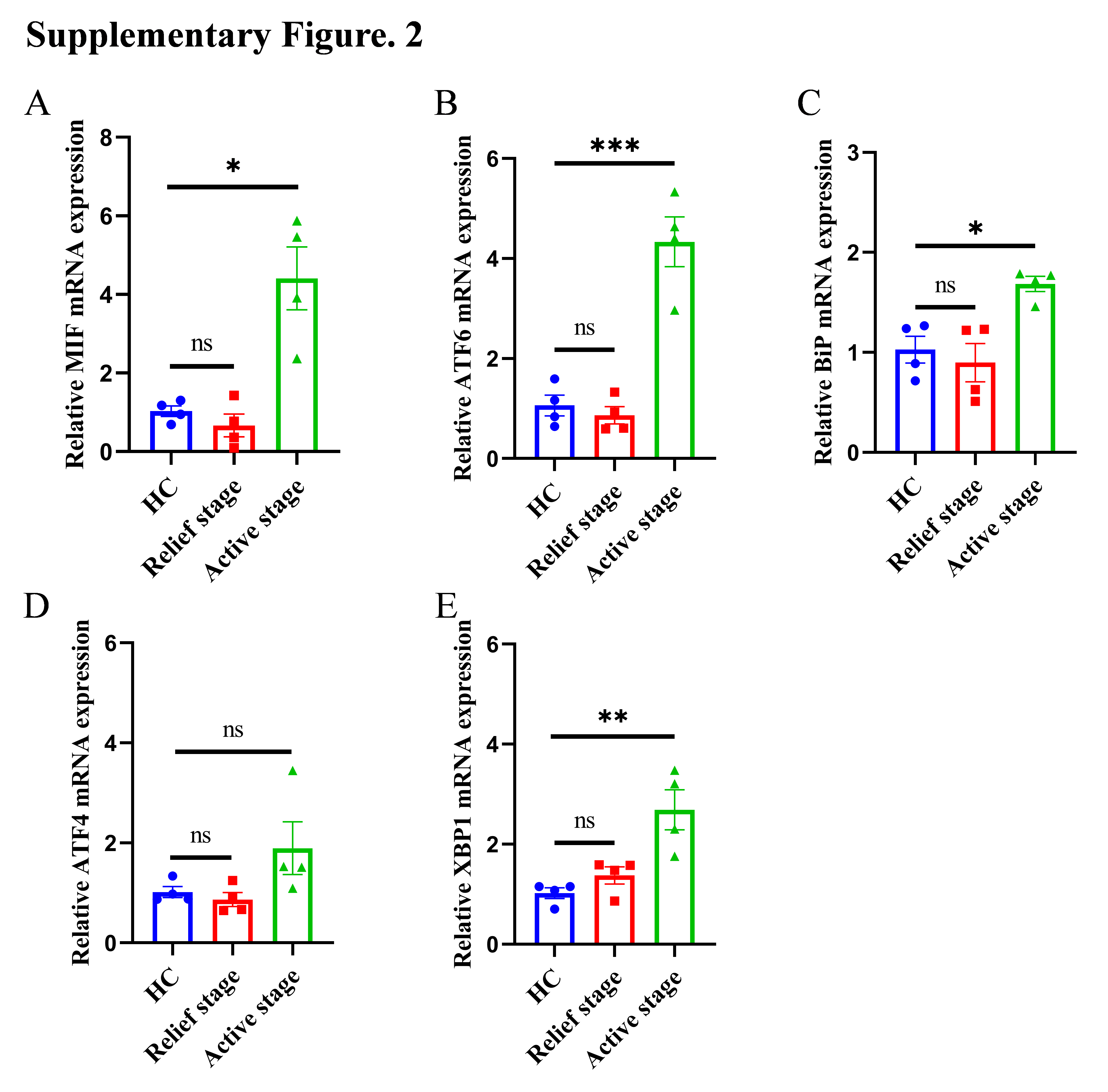

Supplement: Supplementary file 2 — Supplementary Material 2 [file 10020_2024_1005_MOESM2_ESM.tif]
